# Temperature-Induced Sex Differentiation in River Prawn (*Macrobrachium nipponense*): Mechanisms and Effects

**DOI:** 10.3390/ijms25021207

**Published:** 2024-01-19

**Authors:** Gang Jiang, Yucai Xue, Xuxiong Huang

**Affiliations:** 1 Centre for Research on Environmental Ecology and Fish Nutrition (CREEFN) of the Ministry of Agriculture and Rural Affairs, Shanghai Ocean University, Shanghai 201306, China; d210100024@st.shou.edu.cn (G.J.); d220100029@st.shou.edu.cn (Y.X.); 2 Building of China-ASEAN Belt and Road Joint Laboratory on Mariculture Technology and Joint Research on Mariculture Technology, Shanghai 201306, China; 3 National Demonstration Center for Experimental Fisheries Science Education, Shanghai Ocean University, Shanghai 201306, China

**Keywords:** *Macrobrachium nipponense*, temperature-regulating, sex differentiation, sex sensitive phase, regulatory mechanism

## Abstract

*Macrobrachium nipponense* is gonochoristic and sexually dimorphic. The male prawn grows faster and usually has a larger size than the female. Therefore, a higher male proportion in stock usually results in higher yield. To investigate the impact of temperature on sexual differentiation in *M. nipponense*, two temperature treatments (26 °C and 31 °C) were conducted. The results showed that compared to the 31 °C treatment (3.20 ± 0.12), the 26 °C treatment displayed a lower female/male ratio (2.20 ± 0.11), which implied that a lower temperature could induce masculinization in *M. nipponense.* The temperature-sensitive sex differentiation phase was 25–35 days post hatching (DPH) at 26 °C while 15–20 DPH at 31 °C. Transcriptome and qPCR analysis revealed that a lower temperature up-regulated the expression of genes related to androgen secretion, and down-regulated the expressions of genes related to oogonia differentiation. Thirty-one temperature-regulated sex-differentiation genes were identified and the molecular mechanism of temperature-regulated sex differentiation was suggested. The finding of this study indicates that temperature regulation can be proposed as an innovative strategy for improving the culture yield of *M. nipponense*.

## 1. Introduction

The oriental river prawn *Macrobrachium nipponense* (Crustacea; Decapoda; Palaemonidae) is widely distributed in Asian countries [1]. China produced 225,321 tons of *M. nipponense* in 2019, with an estimated value of USD 2.8 billion [2]. Similar to other *Macrobrachium* species, the male oriental river prawn grows faster than the female. The average commercial size of male prawns is 2–2.5 times that of females [1,3]. Consequently, the more male stock in the *M. nipponense* population, the higher the production and profit. In practice, a high proportion of female prawns is often observed in the culture pond of *M. nipponense*, especially during the summer season. Whether this phenomenon is associated with environmental factors remains unclear.

The sex determination of crustaceans occurs in the early development stages [4]. The sex of crustaceans is determined by internal (genetics) and/or external factors (environment) [3,5]. Genetic sex determination (GSD) is determined through the segregation of genes on sex chromosomes, while environmental sex determination (ESD) is influenced by factors such as temperature, nutrition, hormones, and population density [6]. It is reported that GSD and ESD are not mutually exclusive and in some cases act in combination [4,7,8]. For crustaceans, sex differentiation is much more susceptible to the external environment [9,10,11,12]. Temperature is the most widely studied environmental factor in sex differentiation. Many reptiles and fish employ temperature-dependent sex determination (TSD), whereby the temperature regulates the cascade of sex differentiation, promoting undifferentiated gonads developed towards testes or ovaries [4]. Ge et al. [13] and Fisher et al. [14] respectively revealed that sea turtles produced more female offspring at higher temperatures while more male offspring at lower temperatures. Baroiller et al. demonstrated that a high temperature induced masculinization in Nile tilapia [8]. Whether temperature affects the sex differentiation of *M. nipponense* has not been investigated. Furthermore, if the sex differentiation of *M. nipponense* could be induced by temperature, what does the temperature act on? Although several genes have been reported related to sex differentiation in crustaceans, including *IAG*, *Vtg*, *Wnt4*, *CFSH*, *Dmrt* gene family, *Sox* gene family, cell cycle gene family, and *Fem-1*, etc. [15,16,17,18,19,20,21], the TSD-related genes in *M. nipponense* and their mechanism are still unrevealed.

This study aimed to discover the effect and efficiency time of temperature on sex differentiation in *M. nipponense*, as well as the molecular mechanism underlying temperature regulation in this species. This work could help understand the temperature effect on sex differentiation, and provide a potential method for the sex ratio regulation in the *M. nipponense* population.

## 2. Results

### 2.1. The Effect of Temperature on the Sex Ratio of M. nipponense

**Figure 1:**
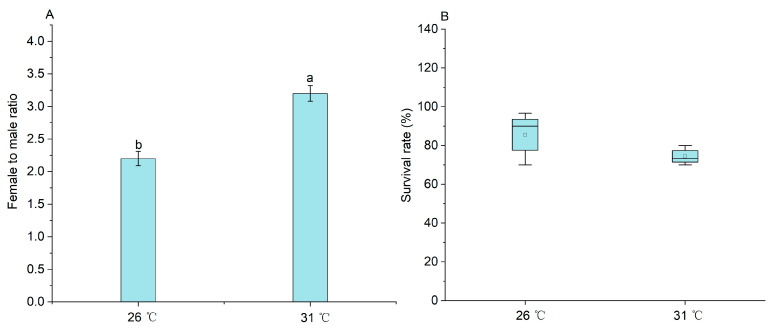
The comparisons on survival and female/male ratio of M. nipponense cultured at 26 °C and 31 °C on 90 DPH. Note: Data are expressed as mean ± SE from triplicates. Different superscript letters indicate significant differences (*p* < 0.05). (**A**) female to male ratio; (**B**) survival rate.

There was a significant difference in the female/male ratio (*p* < 0.05) between these two treatments. The female/male ratio in the 31 °C treatment was 3.20 ± 0.12, whereas, the female/male ratio in the 26 °C treatment was 2.20 ± 0.11 (Figure 1A). No significant difference in the survival rate of *M. nipponense* was observed between the 26 °C and 31 °C treatments at the end of the 90-day culture (*p* > 0.05) (Figure 1B).

### 2.2. Temperature-Sensitive Sex Differentiation Phase of M. nipponense

**Figure 2:**
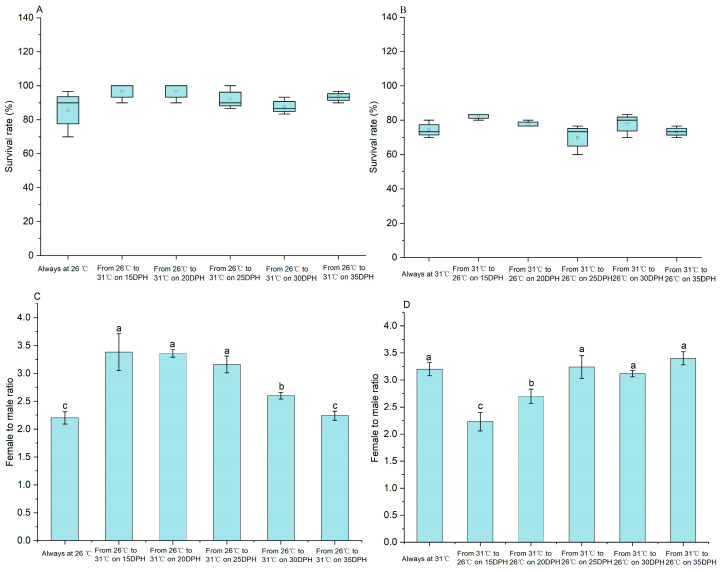
Temperature-sensitive sex differentiation phase of *M. nipponense* cultured at 26 °C and 31 °C. Note: Data are expressed as mean ± SE from triplicates. Different superscript letters indicate significant differences (*p* < 0.05). (**A**) the survival rates on larvae always at 26 °C and the treatments from 26 °C to 31 °C; (**B**) the survival rates on larvae always at 31 °C and the treatments from 31 °C to 26 °C; (**C**) the female to male ratio on larvae always at 26 °C and the treatments from 26 °C to 31 °C; (**D**) the female to male ratio on larvae always at 31 °C and the treatments from 31 °C to 26 °C.

No significant difference in the survival rate of each DPH treatment was observed when compared to the 26 °C treatment at the end of the 90-day culture (*p* > 0.05) (Figure 2A). The 26 °C treatment displayed the lowest female/male ratio (2.20 ± 0.11), orderly followed by the treatment from 26 °C to 31 °C on 35 DPH (2.24 ± 0.08) and the treatment from 26 °C to 31 °C on 30 DPH (2.60 ± 0.06) (Figure 2B). There was no significant difference in the female/male ratio between the 26 °C treatment and the treatment from 26 °C to 31 °C on 35 DPH. The female/male ratio in the treatment from 26 °C to 31 °C on 30 DPH was significantly higher than those of the 26 °C treatment and the treatment from 26 °C to 31 °C on 35 DPH, but significantly lower than those of the treatments from 26 °C to 31 °C on 15, 20, and 25 DPH. There was no significant difference in female/male ratio among the treatments from 26 °C to 31 °C on 15, 20, and 25 DPH (*p* > 0.05). These data demonstrated that the sensitive period on sex differentiation was 25–35 DPH at 26 °C.

Similarly, no significant difference in the survival rate of each DPH treatment was observed when compared to the 31 °C treatment at the end of the 90-day culture (*p* > 0.05) (Figure 2C). There was no significant difference in female/male ratio between the 31 °C treatment and the treatment from 31 °C to 26 °C on 25, 30, and 35 DPH (*p* > 0.05) (Figure 2D). The female/male ratio (2.70 ± 0.13) in the treatment from 31 °C to 26 °C on 20 DPH was significantly higher than that (2.23 ± 0.17) of the treatment from 31 °C to 26 °C on 15 DPH, but significantly lower than that of the treatment from 31 °C to 26 °C on 25 DPH (3.24 ± 0.21). These data demonstrated that the sensitive phase of sex differentiation was 15–20 DPH at 31 °C.

### 2.3. Methylation Levels on M. nipponense from Two Temperature Treatments

**Figure 3:**
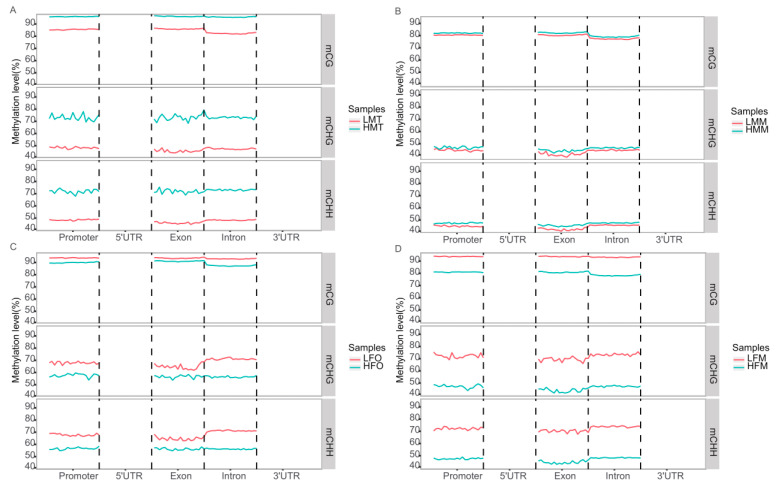
Methylation levels in different tissues of *M. nipponense* cultured at 26 °C and 31 °C on 90 DPH. Note: The abscissa is the functional area classification, and the ordinate is the average methylation rate. (**A**) The methylation rate in the testis of male prawns between 26 °C and 31 °C; (**B**) the methylation rate in the muscle of male prawns between 26 °C and 31 °C; (**C**): the methylation rate in the ovary of female prawns between 26 °C and 31 °C; (**D**): the methylation rate in the muscle of female prawns between 26 °C and 31 °C.

The summary of sequencing data was shown in Tables S2 and S3. The predominant type of mC (methylcystein) in the muscle and gonad of both male and female prawns was mCG, accounting for more than 87.23% of the total mC, orderly followed by the mCHH and mCHG types (Table 1). For the female, the overall methylation levels in the muscle and ovary in the 26 °C treatment were consistently higher by approximately 10% than those in the 31 °C treatment. Figure 3A–D reveal that all methylation regions of the tested tissues occur in the promoter, intron, and exon regions of the gene. For the male, the overall methylation levels in the muscle and testis in the 26 °C treatment were consistently lower by approximately 10% than those in the 31 °C treatment. The results suggested that the male prawns’ gene transcription was partly inhibited in the 31 °C treatment while the female prawns’ gene transcription was partly inhibited in the 26 °C treatment (Figure 3).

### 2.4. DEGs in Male and Female Prawns from Two Temperature Treatments

**Figure 4:**
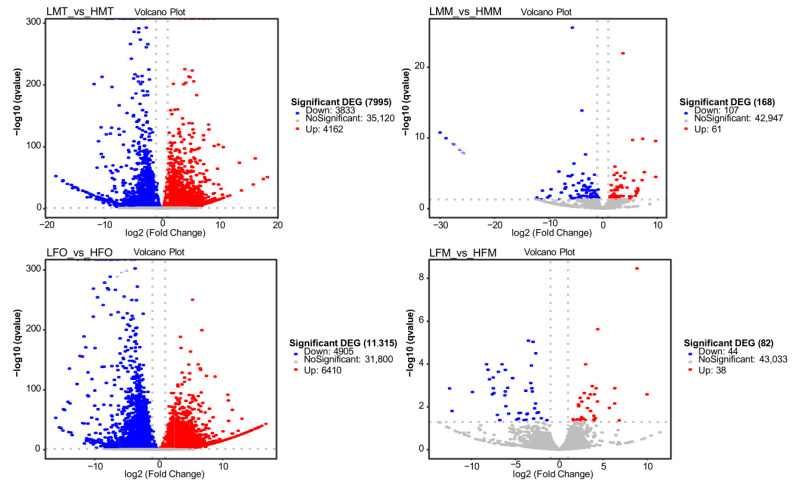
Volcano plot of the DEGs in tissues of *M. nipponense* between 26 °C and 31 °C on 90 DPH. Note: The number of up-regulated, down-regulated, and no significant unigenes are shown in red, blue, and grey, respectively. The *X*-axis represents the comparison value of log 2 (Fold Change), and the *Y*-axis represents the value of |−log 10 (q value)|. LFM: The muscle of female prawns at 26 °C; LMM: The muscle of male prawns at 26 °C; HFM: The muscle of female prawns at 31 °C; HMM: The muscle of male prawns at 31 °C; LFO: The ovary of female prawns at 26 °C; LMT: The testis of male prawns at 26 °C; HFO: The ovary of female prawns at 31 °C; HMT: The testis of male prawns at 31 °C.

The differential expression genes (DEGs) between the 26 °C treatment and the 31 °C treatments were primarily enriched in the gonads of both male and female prawns (Figure 4). A total of 7995 DEGs were identified in the testis, consisting of 3833 down-regulated genes and 4162 up-regulated genes in the 26 °C treatment. Similarly, 11,315 DEGs were identified in the ovary, including 4905 down-regulated genes and 6410 up-regulated genes in the 26 °C treatment. Contrastingly, only 168 DEGs were identified in the muscle of male prawns, comprising 107 down-regulated genes and 61 up-regulated genes in the 26 °C treatment. Likewise, there were only 82 identified DEGs in the muscle of female prawns, consisting of 44 down-regulated genes and 38 up-regulated genes in the 26 °C treatment.

### 2.5. DEG Functional Enrichment Analysis with GO and KEGG Databases

The biological functions of the DEGs, based on the comparisons of the testis, ovary, and muscle between 26 °C and 31 °C, were described using GO and KEGG analyses.

**Figure 5:**
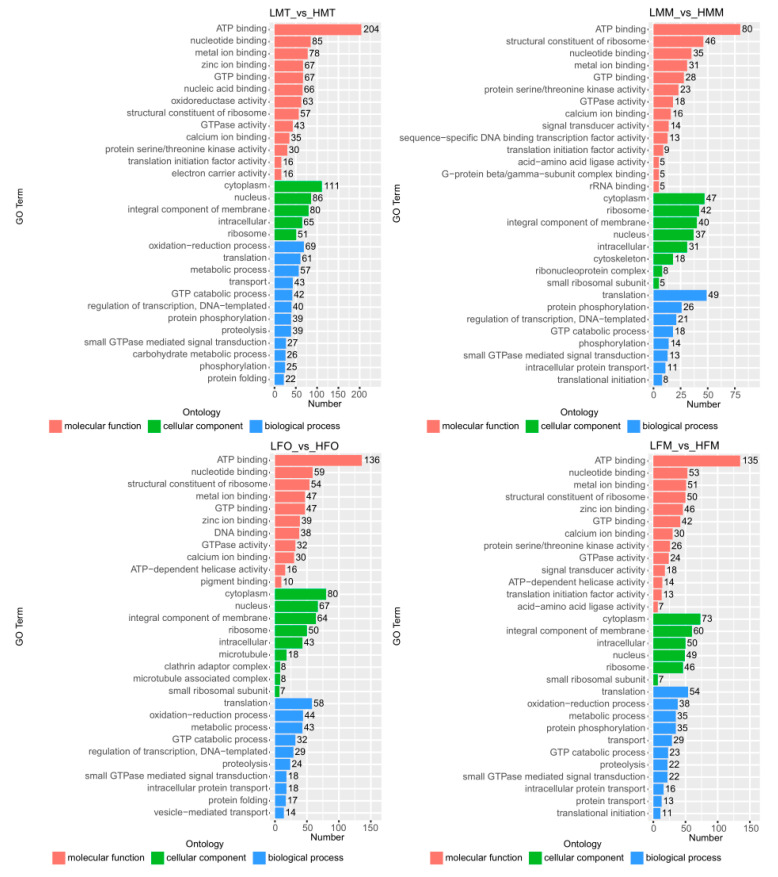
GO classification of DEGs in tissues of *M. nipponense* between 26 °C and 31 °C on 90 DPH. Note: The abscissa is the second-level term under the three categories of GO. The abscissa represents the number of genes annotated to the term; LFM: The muscle of female prawns at 26 °C; LMM: The muscle of male prawns at 26 °C; HFM: The muscle of female prawns at 31 °C; HMM: The muscle of male prawns at 31 °C; LFO: The ovary of female prawns at 26 °C; LMT: The testis of male prawns at 26 °C; HFO: The ovary of female prawns at 31 °C; HMT: The testis of male prawns at 31 °C.

The DEGs in the testis of males at 26 °C vs. 31 °C were mainly involved in ATP binding, cytoplasm, and oxidation-reduction processes, while the DEGs in the muscle and ovary at 26 °C vs. 31 °C were all mainly related to ATP binding, cytoplasm, and translation (Figure 5). These results showed that temperature effectively changed energy metabolism in male and female *M. nipponense*.

**Figure 6:**
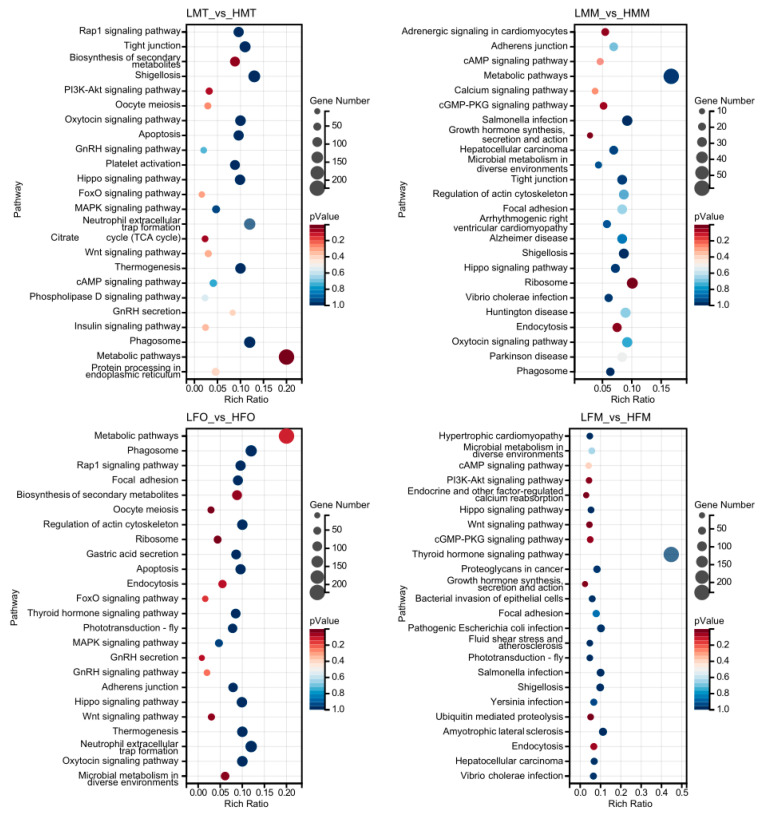
KEGG enrichment of DEGs in tissues of *M. nipponense* between 26 °C and 31 °C on 90 DPH. Note: The ordinate (**left**) is the name of the KEGG signal pathway, the ordinate (**right**) is the number of unigenes annotated to the pathway and the (*p*) value, and the abscissa is the rich ratio. LFM: The muscle of female prawns at 26 °C; LMM: The muscle of male prawns at 26 °C; HFM: The muscle of female prawns at 31 °C; HMM: The muscle of male prawns at 31 °C; LFO: The ovary of female prawns at 26 °C; LMT: The testis of male prawns at 26 °C; HFO: The ovary of female prawns at 31 °C; HMT: The testis of male prawns at 31 °C.

The KEGG analysis revealed that 731, 349, 530, and 333 unigenes were matched known proteins based on DEGs in the testis of males at 26 °C vs. 31 °C, the muscle of males at 26 °C vs. 31 °C, the ovary of females at 26 °C vs. 31 °C, and the muscle of females at 26 °C vs. 31 °C, respectively (Figure 6). The metabolic pathway was the main enriched pathway based on the testis of males at 26 °C vs. 31 °C, the muscle of males at 26 °C vs. 31 °C, and the ovary of females at 26 °C vs. 31 °C, while the thyroid hormone signal pathway was the main enriched pathway based on the muscle of females at 26 °C vs. 31 °C.

### 2.6. Candidates of Temperature-Regulated Sex-Differentiation Genes

To identify the candidate genes of temperature- regulated for *M. nipponense*, a KEGG analysis on the gonads and muscles of male and female prawns at 26 °C versus 31 °C was conducted (Figure S1). Pathways including the “GnRH signaling pathway”, “GnRH Secretion”, “FOXO signaling pathway”, “Wnt signaling pathway”, and “Oocyte meiosis” were identified as potential candidate pathways involved in sex differentiation in *M. nipponense* based on comparisons with published data for other species [22,23,24,25]. Thirty-one DEGs in these pathways, listed in Table 2, were screened out as potential crucial candidate genes of temperature-regulated sex differentiation in *M. nipponense*.

### 2.7. mRNA Expression Profiling of Potential Temperature-Regulated Sex-Differentiation Genes

**Figure 7:**
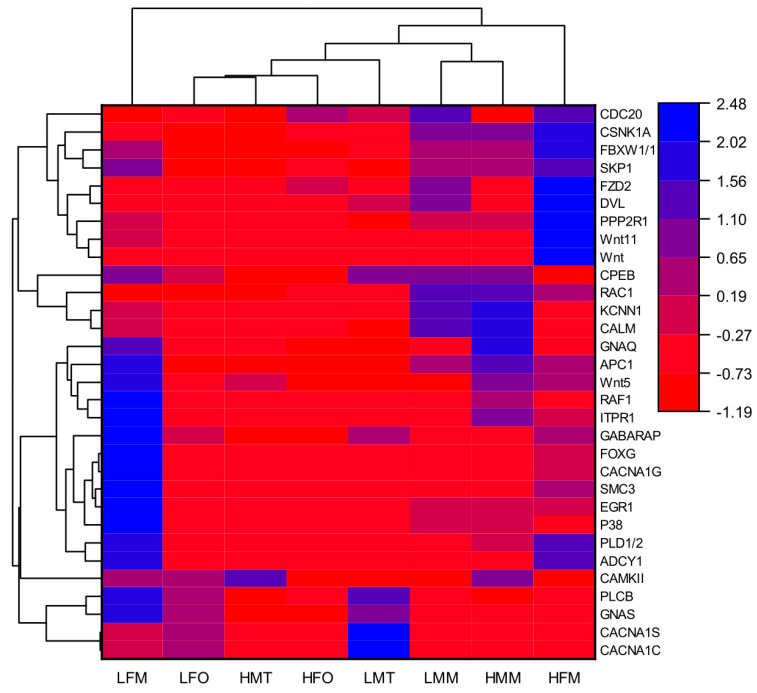
Hierarchical clustering analysis for potential temperature-regulated sex-differentiation genes. Note: The color indicates the log 2-fold change from high (blue) to low (red), as indicated by the color scale. The ordinate (right) is the name of the potential genes, while the abscissa is the name of the tested tissues. LFM: The muscle of female prawns at 26 °C; LMM: The muscle of male prawns at 26 °C; HFM: The muscle of female prawns at 31 °C; HMM: The muscle of male prawns at 31 °C; LFO: The ovary of female prawns at 26 °C; LMT: The testis of male prawns at 26 °C; HFO: The ovary of female prawns at 31 °C; HMT: The testis of male prawns at 31 °C.

The expression profiles on potential temperature-regulated sex-differentiation genes based on cDNA libraries of detected tissues were constructed via complete hierarchical linkage cluster analysis (Figure 7). The results showed a great difference in expression in the tested tissues (especially in muscle), depending on sex and temperature. Furthermore, the expression levels of *CACNA1C*, *CACNA1S*, *GNAS*, *PLCB*, *CAMKII*, and *ADCY1* in the males’ testis and muscle in the 26 °C treatment were significantly higher than those in the 31 °C treatment, suggesting that these genes may play a significant role in the development of male prawns.

### 2.8. Quantitative Polymerase Chain Reaction (qPCR) Analysis

**Figure 8:**
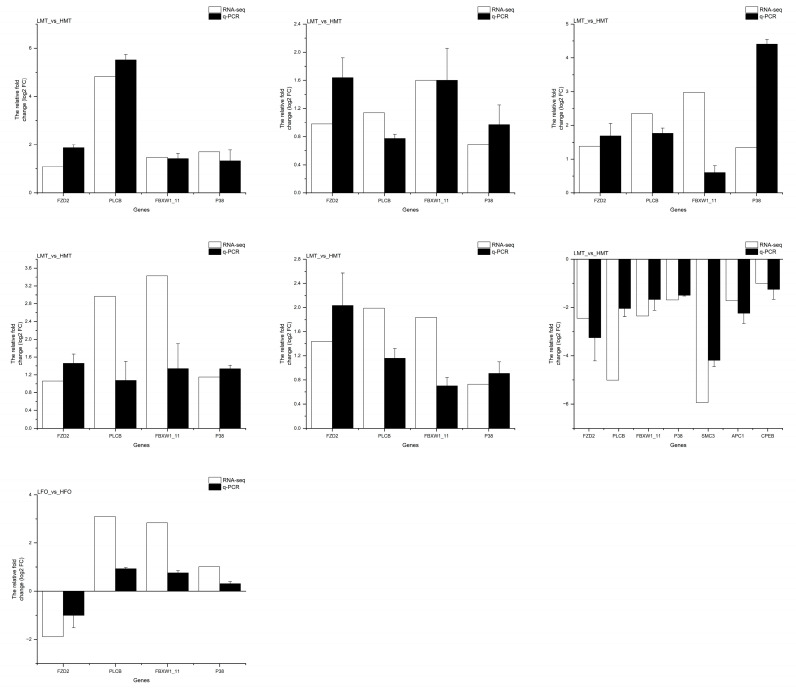
Comparisons of the qRT-PCR and RNA-seq of potential temperature-regulated sex-differentiation genes. Note: The qRT-PCR and RNA-seq results are shown in black and white, respectively. The abscissa presents the name of DEGs; the ordinate shows the expression levels. Data are expressed as mean ± SE from triplicate groups. LFO: The ovary of female prawns at 26 °C; LMT: The testis of male prawns at 26 °C; HFO: The ovary of female prawns at 31 °C; HMT: The testis of male prawns at 31 °C.

The transcriptomic data were validated with qRT-PCR. The expression patterns of all these 31 DEGs in adults matched mutually between qRT-PCR and RNA-seq (Figure 8).

Subsequently, qRT-PCR analysis was employed to further evaluate the expression levels of the DEGs in larvae from 15 DPH to 35 DPH in the treatments always at 26 °C and 31 °C (Figure S2).

At 26 °C, the relative expressions of 16 DEGs, such as *CACNA1G*, *CACNA1S*, *CALM*, *CAMKII*, *CPEB*, *FOXG*, *GABARAP*, *KCNN1*, *P38*, *PLCB*, *PLD1/2*, *RAF1*, *CACNA1C*, *PPP2R1*, *WNT11*, and *WNT5*, first increased and then decreased from 25 DPH to 35 DPH, and peaks appeared on 30 DPH which were significantly higher than those on 15 DPH and 20 DPH (*p* < 0.05). On the other hand, the relative expressions of the other 13 DEGs, including *ADCY1*, *APC1*, *CDC20*, *FBXW1/11*, *ITPR1*, *SKP1*, *SMC3*, *CSNK1A*, *DVL*, *GNAQ*, *RAC1*, *EGR1*, and *FZD2*, decreased from 25 DPH to 35 DPH, and peaks appeared on 25 DPH (except *EGR1*) which were also significantly higher than those on 15 and 20 DPH (*p* < 0.05). Furthermore, the relative expressions of the genes, including *WNT11* and *GNAS*, showed no significant difference (*p* > 0.05) from 25 DPH to 35 DPH, but were significantly lower than those on 15 DPH and 20 DPH (*p* < 0.05).

At 31 °C, the relative expressions of 25 DEGs, such as *CACNA1G*, *CACNA1S*, *CALM*, *CAMKII*, *CDC20*, *CPEB*, *EGR1*, *FBXW1/11*, *FOXG*, *GABARAP*, *ITPR1*, *KCNN1*, *PLCB*, *PLD1/2*, *SMC3*, *WNT11*, *CSNK1A*, *RAC1*, *SKP1*, *DVL*, *ADCY1*, *SKP1*, *WNT*, *CACNA1C*, and *FZD2*, decreased from 15 DPH to 20 DPH, and peaks appeared on 15 DPH (except *ADCY1*) which were significantly higher than those on 25 DPH, 30 DPH, and 35 DPH (*p* < 0.05). On the other hand, the relative expressions of the other four DEGs, including *APC1*, *P38*, *RAF1*, and *PPP2R1*, increased from 15 DPH to 20 DPH. Furthermore, the relative expressions of the remaining two DEGs, including *GNAQ* and *GNAS*, showed no significant difference from 15 DPH to 35 DPH (*p* > 0.05).

## 3. Discussion

GSD is typically governed by sex chromosomes, which exhibit two main types: male heterogamety (XY males and XX females) and female heterogamety (ZZ males and ZW females) [26,27]. If the sex determination of an organism is absolutely GSD, generally the female/male ratio in the offspring is 1:1. In this study, both the female/male ratios at 26 °C and 31 °C deviated from 1:1. This demonstrated that sex differentiation in *M. nipponense* is not only determined by genetics but also by ESD. There was a significant difference in the female/male ratio between the 26 °C and 31 °C treatments. This implied that sex differentiation in *M. nipponense* is susceptible to temperature. Lower temperatures could induce masculinization in *M. nipponense*. Similar results were also reported in domestic chicken (*Gallus domesticus*) [28], sea turtles [29], and European sea bass (*Dicentrarchus labrax* L.) [30], while lower temperatures induced more female individuals in other species such as Nile tilapia (*Oreochromis mossambicus*) [8], *Drosophila melanogaster*, and *Caenorhabditis elegans* [31]. The detailed effect of temperature on sex determination in ESD organisms is species-specific.

Sex determination is not a point process but rather spans several days during development [32]. The phase in which temperature influences the sexual phenotype of the gonad is referred to as the sensitive period for sex differentiation [33]. Thompson-Davis revealed that culturing the *G. domesticus* larvae on 22 DPH to 24 DPH at 22 °C induced more male individuals [28]. Rougeot et al. also demonstrated that culturing *O. mossambicus* from 10 DPH to 28 DPH at higher temperatures increased the male ratios in a population [34]. Therefore, ascertaining the temperature-sensitive gonadal differentiation phase is crucial for manipulating sex via temperature. Jin et al. reported that the formation of the testis, ovary, and androgenic gland in *M. nipponense* occurs on PL10 to PL22, much faster than those in other aquatic animals [35,36]. Some factors, such as age, individual size, and external environments influence individual development [37]. The temperature-sensitive period on sex differentiation in *M. nipponense* was temperature-depended. The temperature-sensitive period on sex differentiation in *M. nipponense* was 25–35 DPH at 26 °C and 15–20 DPH at 31 °C, respectively.

Recently, there has been evidence supporting the role of environmental cues, such as temperature, in regulating sex differentiation through changes in DNA methylation [38]. For instance, Ge et al. discovered that certain genes related to sex differentiation were hypermethylated at higher temperatures, leading to transcriptional silencing and ultimately causing sex reversal in turtles [13]. Similarly, Matsumoto et al. found that temperature, especially the production temperature of females, caused demethylation in the cyp19a promoter region. This resulted in the temperature-specific expression of cyp19a, promoting female differentiation [39]. Epigenetic mechanisms, activated by temperature, can lead to varying levels of DNA methylation in promoters associated with gonadal development. These changes in DNA methylation subsequently impact gene expression, specifically estrogen synthesis, which drives natural sex changes [40]. In this study, we observed differences in DNA methylation levels between males and females in the gonad and muscle of *M. nipponense*. Similar results have also been reported in buffalo research [41]. Additionally, we found that the DNA methylation level in the testis and muscle of male prawns was higher at 31 °C than at 26 °C. Conversely, the DNA methylation level in the ovary and muscle of female prawns was higher at 26 °C than at 31 °C. It is speculated that a higher temperature may hinder male-related gene transcription, while a lower temperature may hinder female-related gene transcription. As a result, at higher temperatures, the population displayed a higher female proportion, and at lower temperatures, it displayed a higher male proportion. However, due to the lack of CPG island information in the genome of *M. nipponense*, we could not focus on which genes were methylated by temperature regulation. Therefore, other omics methods are needed to reveal the possible mechanism of temperature-regulated sex differentiation in this species.

Gonadal differentiation is a complex regulatory process that involves a network of genes [42,43]. Within this network, the terms of gene activation timing could be changed [44], and gene positions could also be changed in the regulatory pathways between roles as master regulators or downstream actors [45]. These changes affect the final differentiation of gonads. The current study found that compared to 31 °C treatments, the DEGs of the testis and ovary from 26 °C treatments were enriched in some signaling pathways related to sex differentiation (Figure 6), including the “GnRH signaling pathway”, “GnRH Secretion”, “FOXO signaling pathway”, “Wnt signaling pathway”, and “Oocyte meiosis”. The DEGs involved in these signaling pathways may be crucial potential genes in regulating the temperature-sensitive sex differentiation of *M. nipponense*.

Hormones are the primary communicators between external conditions and physiological activities, as environmental stimulants must first be converted into physiological signals to affect sex differentiation [46]. The GnRH signaling pathway plays a central role in hormone secretion systems [47]. Du et al. verified that *M. nipponense* has the same GnRH signaling pathway as vertebrates [48]. In this study, the screened GnRH signaling pathway from KEGG analysis may support the hypothesis that the GnRH signaling pathway participating in the regulation of sex differentiation in *M. nipponense* is likely similar to that of vertebrates. RNA-seq discovered that eight DEGs of the GnRH signaling pathway, including voltage-dependent calcium channel L type alpha-1C (*CACNA1C*), guanine nucleotide-binding protein G(s) subunit alpha (*GNAS*), calcium/calmodulin-dependent protein kinase (CaM kinase) II (*CAMK II*) adenylate cyclase 1 (*ADCY1*), p38 MAP kinase (*P38*), early growth response protein 1 (*EGR1*), and phospholipase D1/2 (*PLD1/2*), up-regulated in the testis at 26 °C vs. 31 °C. This discovery indicated that these genes may play crucial roles in testis development at 26 °C. The *CACNA1C* gene has been identified as a crucial component of the GnRH signaling pathway in vertebrates. The *CACNA1C* gene is synergically involved in ERK activation and leads to stimulating the secretion of the follicle-stimulating hormone (*FSH*) and luteinizing hormone (*LH*) [49]. *LH* targets testicular stromal cells to stimulate testosterone (T) production, while *FSH* can promote the development of the epithelium and secondary spermatocytes within the ducts, leading to cooperation with *LH* for complete spermatogenesis and the maturation of sperm [50]. The present study revealed that the *CACNA1C* gene exhibited a higher expression level during the sex-differentiation phase at 26 °C than at 31 °C. It was speculated that a lower temperature up-regulated *CACNA1C* expression, which facilitates the differentiation of primordial germ cells into male sperm cells. Consequently, the population at 26 °C likely exhibited a higher proportion of male prawns than that at 31 °C. *EGR-1* also plays an important role in *LH* expression [51]. Therefore, the improved *EGR-1* expression is also beneficial to the male differentiation of *M. nipponense*. Tourtellotte et al. demonstrated that the regulation of *EGR* in cell growth and differentiation was taking place at the early meiotic stage in male mice [52]. In this study, the peak expression of the *EGR1* in the prawns occurred on 20 DPH at 26 °C and 15 DPH at 31 °C, respectively.

In mammals, the inactivation of sex-related genes leads to the masculinization of female gonads, whereas over-expression leads to the feminization of male gonads [53,54]. The Wnt signaling pathway appears to be implicated in female gametogenesis. It plays complementary roles in ovary determination in mice, including the activation of ovarian genes and the inhibition of testis formation [55]. In crustaceans, such as the *Scylla Paramamosain*, Farhadi et al. have also demonstrated the significance of the WNT gene in ovarian development [56]. Rhen reported that temperature-changing gene expression is involved in Wnt signaling [57]. In the current study, the participants of the Wnt signaling pathway, including wingless-type MMTV integration site family, member 1 (*WNT*), wingless-type MMTV integration site family, member 5 (*WNT5*), wingless-type MMTV integration site family, member 11 (*WNT11*), segment polarity protein dishevelled (*DVL*), Frizzled 2 (*FZD2*), phosphatidylinositol phospholipase C, beta (*PLCB*), Ras-related C3 botulinum toxin substrate 1 (*RAC1*), S-phase kinase-associated protein 1 (*SKP1*), and casein kinase 1, alpha (*CSNK1A*), were screened out as DEGs. The RNA-seq and qPCR analysis showed that the *Wnt* and *Wnt 11* genes were down-regulated at 26 °C versus 31 °C. We speculated that the Wnt pathway could act on gonadal differentiation in *M. nipponense*, and the up-regulated *Wnt* and *Wnt 11* genes appear to hurt male sex differentiation in *M. nipponense*. WNTs interact with FZDs through their cysteine-rich domain (*CRD*) 5 at the N terminus of the receptors, leading to the initiation of distinct downstream signaling pathways [58]. This study revealed that the *FZD2* and *DVL* genes were down-regulated significantly at 26 °C versus 31 °C during the sex-sensitive phase. The expression of the *SKP1* gene in larvae reached its peak on 15 DPH at 31 °C and 25 DPH at 26 °C, respectively. As the larvae developed, the expression of the *SKP1* gene was consistently higher at 31 °C than at 26 °C. This suggests that lower temperatures could inhibit the expression of the *SKP1* gene. Guan et al. have demonstrated that the *SKP1* gene plays a role in regulating the degradation of sex hormone receptors in mouse cells, as well as maintaining a stable level [59]. Therefore, we speculated that temperature could regulate the *SKP1* expression, and impact the secretion of intracellular sex hormone levels, finally leading to the biological outcome of sex differentiation. Compared to that at 31 °C, the prawns at 26 °C displayed up-regulated RAC1 in the ovary but no change in the testis, while the *PLCB* gene was down-regulated in the ovary but not changed in the testis. Abu Risha et al. [60] also demonstrated similar results. These findings indicated a cross-talk of the genes in the Wnt signaling under thermal stress.

The FOXO signaling pathway was notably enriched from the transcripts, where forkhead box protein G (*FOXG*), p38 MAP kinase (*P38*), and GABA(A) receptor-associated protein (*GABARAP*) genes were enriched in this pathway. Compared to those at 31 °C, the RNA_seq revealed that *FOXG* was up-regulated in the testis but down-regulated in the ovaries at 26 °C. Jin et al. have reported that the expression of the *Fox2* gene in male *M. nipponense* was significantly higher than that in females, and the up-regulation of *Fox2* may promote male differentiation and development in *M. nipponense* [61]. Thia suggests that *FOXG*, similar to *Fox2*, likely plays a positive role in promoting male differentiation in *M. nipponense*. The qPCR analysis on the sex-differentiation phase of *M. nipponense* revealed a significant up-regulation of the *FOXG* gene at 26 °C versus 31 °C. These results provide evidence on the relationships between temperature and terms of gonadal fate in *M. nipponense*. Lower temperatures can promote the expression of *FOXG* and finally induce male differentiation. Like the *FOXG* expression profile, the expression of the *p38* gene was also up-regulated in the testis at 26 °C versus 31 °C. This demonstrated that *p38* is important for testis development. Luo et al. also confirmed this view, suggesting that *p38* plays a vital role in the somatic cells of the spermatozoa as they proliferate and mature to form sperm [62]. In addition, the p38 gene was also screened out in the GnRH signaling pathway, further suggesting its positive role in promoting the male differentiation of *M. nipponense*. The lower temperature also promote the expression of the *GABARAP* gene in the testis, while the qPCR analysis demonstrated that the expression of the *GABARAP* gene was notably higher at 31 °C versus 26 °C on 15 DPH, followed by a subsequent reversal in trend. The proteins encoded by the *GABARAP* gene participate in various cellular physiological processes, such as autophagy, membrane transport, and the regulation of receptor stability [63,64]. Autophagy, a fundamental cellular metabolic process responsible for eliminating damaged or aged components from cells, plays a crucial role in maintaining a stable intracellular environment [65,66]. Notably, the secretion and regulation of sex hormones are intricately associated with autophagy [67]. Therefore, we hypothesized that a lower temperature may enhance the expression of the *GABARAP* gene in larvae, thereby augmenting the role of autophagy in vivo and ultimately impacting the secretion of sex hormones.

Twenty-one DEGs exhibited different expression profiles in the testis from the oocyte meiosis signaling pathway based on 26 °C versus 31 °C, among which only one was up-regulated. As the foundation of sexual reproduction, meiosis directly affects the formation of gametes and the character and sex of individual offspring [23]. Pennell et al. revealed that due to the fact that male gametes tend to require less nutrition and a heavy chromosome count, in most cases, enhanced oocyte meiosis promotes female differentiation [68,69,70]. Bull et al. also reported that high temperatures could enhance estrogen secretion and meiosis rate [71,72]. Therefore, the lower temperature could inhibit oocyte meiosis. This may demonstrate that the population at 26 °C likely exhibited a higher proportion of male prawns than that at 31 °C.

**Figure 9:**
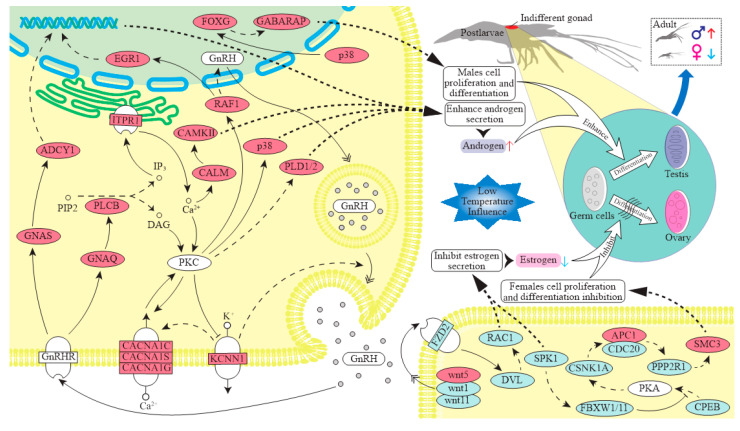
Hypothesized scheme of the temperature regulation process on sex differentiation in *M. nipponense.* Note: Solid lines mean confirmed effects and dotted lines mean possible effects. The red, blue, and white ovals represent up-regulated, down-regulated, and un-differentially expressed genes, respectively.

Based on the analysis of the key pathways and candidate genes, a possible molecular mechanism of temperature-regulated sex differentiation in *M. nipponense* was constructed (Figure 9). In a lower temperature environment, *KCNN1* is stimulated and transmits signals to activate the GnRH signaling pathway, leading to the up-regulation of the genes *CACNA1C*, *CACNA1S*, and *CACNA1G* that transmit signals to the nucleus. Subsequently, the downstream genes *CALM*, *p38*, *PLD1/2*, and *EGR1* are significantly expressed. Furthermore, the activation signal from GnRH promotes the expression of the downstream gene *ADCY1* through its receptor *GnRHR*. These changes in gene expression result in an increased secretion of androgenic hormones, which leads to germ cell differentiation into spermatogonia and subsequent sperm development. Ultimately, this process increases the proportion of male prawns in the population under lower temperatures. Additionally, in lower temperature conditions, the genes involved in oocyte meiosis are down-regulated, potentially reducing estrogen secretion and preventing oocyte differentiation. This ultimately leads to a decrease in the proportion of female prawns in the population. Conversely, a decrease in temperature positively affects the FOXO signaling pathway by activating the *P38* gene and facilitating the expression of *FOXG*, resulting in an increased secretion of androgenic hormones in post-larvae which leads to germ cell differentiation into spermatogonia and subsequent sperm development. A temperature decreasing results in the down-regulation of *Wnt* and *Wnt11*, which inhibits the interaction between WNTs and FZDs, leading to a hindrance to female germ cell induction and female prawn production. Overall, we speculated that sex hormones may be the link between temperature and the sex-determining in *M. nipponense*. Temperature regulates the expression of these genes, which affects the secretion of sex hormones in *M. nipponense*, ultimately resulting in variations in the sex ratio.

## 4. Materials and Methods

### 4.1. Animals

The broodstocks of *M. nipponense* (5 males, BW 3.30 ± 0.18 g; 10 females, BW 0.89 ± 0.03 g) were used in this study. They were established based on inbred offspring from a monophyletic sibling, as described by Jiang et al. [73].

### 4.2. Sex Differentiation under Different Temperatures

The newly hatched zoea from a berried female were divided into two 5-L polyethylene tanks equally and cultured at low (26 ± 0.5 °C) and high (31 ± 0.5 °C) temperatures, respectively. Once the zoea completely metamorphosed into post-larvae, 90 healthy seeds in each temperature treatment were randomly allocated into three aquariums (30 cm × 30 cm × 30 cm) for a further 90-day culture at the original temperature with aquatic plant (*Ceratophyllum demersum* L.) shelter, enough diet, and continuous aeration. At the end of the trial, the survivals in each replicate were counted and the gender of the prawns was distinguished via morphological features under an anatomical microscope.

Adult male (BW 1.42 ± 0.05 g) and female (BW 0.53 ± 0.12 g) prawns from the 31 °C and 26 °C treatments were collected and anesthetized on ice for 5 min before being sacrificed by dissection. The muscle, testis, and ovary were sampled, respectively, and immediately immersed in liquid nitrogen. To ensure sufficient amounts of DNA and RNA samples, three gonad tissues were pooled to form one biological replicate, and three replicates were sequenced for both males and females at each temperature treatment.

### 4.3. Temperature-Sensitive Sex-Differentiation Phase

To investigate the temperature-sensitive sex-differentiation phase of *M. nipponense* at different temperatures, zoea hatched from berried females were allocated into two 10-L polyethylene tanks and cultured at low (26 ± 0.5 °C) and high (31 ± 0.5 °C) temperatures, respectively. Then, a total of 90 healthy larvae (triplicates) were randomly collected on the 15, 20, 25, 30, and 35 DPH from each temperature treatment. These three triplicates were then subjected to a switch from the initial temperature treatment (26 °C or 31 °C) to the alternated temperature treatment (31 °C or 26 °C) for continuous culture until the prawns were adult. At the end of the trial, the survivals in each replicate were counted and the gender of the prawns was distinguished.

### 4.4. Whole-Genome Bisulfite Sequencing (WGBS) Libraries

Genomic DNA was extracted using the Magnetic Universal Genomic DNA Kit (TIANGEN, Beijing, China). The quality was analyzed using NanoDrop 2000 (Thermo Scientific, Waltham, MA, USA). The OD260/280 and concentration should range from 1.8 to 2.0 and >200 ng/μL, respectively, so that they can be used for sequencing. The genomic pools were fragmented to an average size of 250 bp using sonication. Then, the fragments were subjected to DNA-end repair, dA addition at the 3′ end, and the ligation of sequencing adaptors and index for bisulfite treatment with the Zymo EZ DNA Methylation-Gold kit (Zymo Research, Irvine, CA, USA). With the Pippin-Prep platform (Sage Science, Beverly, MA, USA), fragments of a target size of 300–350 bp were selected for the construction of the WGBS libraries using the TruSeq DNA Methylation Kit from Illumina. The 2 × 100 bp paired-end reads were generated using Illumina HiSeq-2000 (Illumina, San Diego, CA, USA).

### 4.5. Transcriptomic Analysis Using RNA-Sequencing

The RNA pools of muscle and gonads from males and females were extracted using TRIzol (Life Technologies, Carlsbad, CA, USA). The quantity of total RNA was determined with a Qubit fluorometer (Life Technologies) and assessed by measuring RINs with a Bioanalyzer Chip RNA 7500 series II (Agilent, Santa Clara, CA, USA). The OD260/280 and RIN should range from 1.8 to 2.0 and 7.0 to 10.0, respectively, to ensure the purity of the RNA samples. Approximately 1 μg of each RNA pool was used to prepare an mRNA-Seq library with the NEBNext UltraTM RNA Library Prep Kit for Illumina (NEB, Ipswich, MA, USA). The libraries with a fragment length of preferentially 240bp were purified with the AMPure XP system (Beckman Coulter, Beverly, MA, USA), and library quality was assessed on the Agilent Bioanalyzer 2100 system. The libraries for male and female prawns were sequenced using Hiseq 2000 (Illumina).

The clean reads were mapped to the *M. nipponense* genome [17]. The software Cufflinks (version 2.2.1) [74] normalized gene expressions to the quantified transcription levels (FPKM; fragments per kilobases per million). The DEGs (differentially expressed genes) were calculated using R package edgeR [75]. The Benjamini–Hochberg correction method was used to correct the significance of the p-value of the original test hypothesis to obtain the false discovery rate (FDR) [76]. A fold change was used to present the expression ratio between the comparison groups. An FDR < 0.05 was considered as the differentially expressed genes (DEGs), log_2_^foldchange^ ≥ 1 was up-regulated, and log_2_^foldchange^ ≤ −1 was down-regulated. The DEGs were used for functional enrichment analysis, such as the Kyoto Encyclopedia of Genes and Genomes (KEGG) pathway and gene ontology (GO) terms analysis.

### 4.6. Quantitative Real-Time PCR (qRT-PCR) Analysis

qRT-PCR was used to measure the relative mRNA expression of 31 DEGs in adult male and female prawns, which were always cultured at a water temperature of 26 °C and 31 °C. Independent RNA samples from testis and ovaries were extracted using Trizol reagent (Sangon, Shanghai, China), and the cDNA was synthesized using the PrimeScriptTM RT Reagent Kit with gDNA Eraser (Takara, Shanghai, China). Agilent AriaMx Real-Time PCR System (Agilent) was used to carry out the SYBR Green qRT-PCR assay. The expression levels of the candidate DEGs were further quantified in the larvae on 15, 20, 25, 30, and 35 DPH in the 26 °C and 31 °C treatments, to screen out the DEGs which were candidates of temperature-regulating sex-differentiation genes in *M. nipponense*. Five larvae were pooled to form one biological replicate to avoid randomness. Three biological replicates were collected for each sample.

The procedures and analysis methods were according to our previous study [73], using the Primer5 software (version 5.0) designed primers [77] and ß-actin as an internal control (Table S1).

### 4.7. Statistical Analysis

All data are presented as means ± standard error. The statistical analysis was performed with an analysis of variance (SPSS 25.0). An independent sample *t*-test was used to test the survival and the female-to-male ratio; one-way ANOVA and Duncan’s new multiple-range test was used to test the female-to-male ratio on different transferring DPH in the 26 °C and 31 °C treatments, and the relative expression of temperature-regulated sex-differentiation genes between the 26 °C treatment and the 31 °C treatment. Differences between treatments were considered significant when *p* < 0.05.

## 5. Conclusions

This research demonstrated an increasingly complex sex-determined system in *M. nipponense,* including the effects of temperature, as well as possible genetic and epigenetic factors. Candidate genes associated with sex differentiation and the temperature-sensitive sex differentiation phase were identified. Additionally, the possible molecular regulation mechanism on temperature-regulated sex differentiation in *M. nipponense* was put forward. These findings have important implications for understanding the genetics associated with the environmental regulation of sex differentiation in *M. nipponense*.

## Figures and Tables

**Table 1:** Distribution of mC types in different tissues of *M. nipponense* cultured at 26 °C and 31 °C on 90 DPH.

Category	LFM	HFM	LMM	HMM	LFO	HFO	LMT	HMT
mCG	91.97 ± 0.18 ^a^	92.66 ± 0.18 ^a^	92.09 ± 0.11 ^a^	89.78 ± 1.01 ^a^	87.23 ± 1.11 ^a^	90.49 ± 0.38 ^a^	89.66 ± 0.41 ^a^	92.25 ± 0.15 ^a^
mCHG	1.92 ± 0.04 ^c^	1.93 ± 0.04 ^c^	2.04 ± 0.03 ^c^	2.47 ± 0.18 ^c^	2.56 ± 0.23 ^c^	2.19 ± 0.07 ^c^	2.36 ± 0.08 ^c^	1.77 ± 0.03 ^c^
mCHH	6.29 ± 0.14 ^b^	5.41 ± 0.14 ^b^	5.87 ± 0.08 ^b^	7.75 ± 0.83 ^b^	8.48 ± 0.88 ^b^	7.32 ± 0.31 ^b^	7.99 ± 0.34 ^b^	5.98 ± 0.12 ^b^

Note: Data are expressed as mean ± SE from triplicate groups. Different superscript letters indicate significant differences (*p* < 0.05). LFM: The muscle of female prawns at 26 °C; LMM: The muscle of male prawns at 26 °C; HFM: The muscle of female prawns at 31 °C; HMM: The muscle of male prawns at 31 °C; LFO: The ovary of female prawns at 26 °C; LMT: The testis of male prawns at 26 °C; HFO: The ovary of female prawns at 31 °C; HMT: The testis of male prawns at 31 °C. mCG: Methylation at CG site; mCHG: Methylation at CHG site; mCHH: Asymmetrical methylation of CHH sites.

**Table 2:** Temperature affects genes related to sex differentiation in *M. nipponense*.

Signal Pathway	DEGs	Gene Annotation	Tissues	
			LMT_vs_ HMT	LFO_vs_ HFO
	*CACNA1C*	voltage-dependent calcium channel L type alpha-1C	Up	No
GnRH signaling pathway	*GNAS*	guanine nucleotide-binding protein G(s) subunit alpha	Up	No
	*CAMKII*	calcium/calmodulin-dependent protein kinase (CaM kinase) II	Up	Down
	*ADCY1*	adenylate cyclase 1	Up	Down
	*P38*	p38 MAP kinase	Up	No
	*EGR1*	early growth response protein 1	Up	No
	*CALM*	calmodulin	Up	Down
	*PLD1_2*	phospholipase D1/2	Up	Down
GnRH Secretion	*ITPR1*	inositol 1,4,5-triphosphate receptor type 1	Up	Down
	*KCNN1*	potassium intermediate/small conductance calcium-activated channel subfamily N member 1	Up	No
	*CACNA1G*	voltage-dependent calcium channel T type alpha-1G	Up	No
	*PLCB*	phosphatidylinositol phospholipase C, beta	Down	No
	*RAF1*	RAF proto-oncogene serine/threonine-protein kinase	Up	Down
	*CACNA1S*	voltage-dependent calcium channel L type alpha-1S	Up	No
	*GNAQ*	guanine nucleotide-binding protein G(q) subunit alpha	Up	Down
FOXO signaling pathway	*FOXG*	forkhead box protein G	Up	No
	*P38*	p38 MAP kinase	Up	No
	*GABARAP*	GABA(A) receptor-associated protein	Up	No
Wnt signaling pathway	*Wnt*	wingless-type MMTV integration site family, member 1	Down	No
	*Wnt5*	wingless-type MMTV integration site family, member 5	Up	Down
	*Wnt11*	wingless-type MMTV integration site family, member 11	Down	No
	*DVL*	segment polarity protein dishevelled	Down	Up
	*FZD2*	Frizzled 2	Down	Up
	*PLCB*	phosphatidylinositol phospholipase C, beta	No	Down
	*RAC1*	Ras-related C3 botulinum toxin substrate 1	No	Up
	*SKP1*	S-phase kinase-associated protein 1	Down	Up
	*CSNK1A*	casein kinase 1, alpha	Down	Up
Oocyte meiosis	*FBXW1/11*	F-box and WD-40 domain protein 1/11	Down	Up
	*CPEB*	cytoplasmic polyadenylation element-binding protein	Up	No
	*APC1*	anaphase-promoting complex subunit 1	Up	Down
	*CDC20*	cell division cycle 20, cofactor of APC complex	Down	Up
	*SMC3*	structural maintenance of chromosome 3 (chondroitin sulfate proteoglycan 6)	Up	No
	*PPP2R1*	serine/threonine-protein phosphatase 2A regulatory subunit A	Down	No

Note: LFO: The ovary of female prawns at 26 °C; LMT: The testis of male prawns at 26 °C; HFO: The ovary of female prawns at 31 °C; HMT: The testis of male prawns at 31 °C.

## Data Availability

Curated versions of the genomes were assembled, annotated, and analyzed during the current study based on the *M. nipponense* genome (https://gigadb.org/dataset/100843) (accessed on 11 April 2022). The reads of *M. nipponense* transcriptome were submitted to NCBI with the accession number PRJNA9878611.

## Supplementary Materials

The following supporting information can be downloaded at: https://www.mdpi.com/article/10.3390/ijms25021207/s1.
