# Supplementary material for: Temperature-Induced Sex Differentiation in River Prawn (Macrobrachium nipponense): Mechanisms and Effects
Source: Int J Mol Sci. 2024 Jan 19;25(2):1207. doi: 10.3390/ijms25021207 (PMC10816446; doi:10.3390/ijms25021207)
Supplement: Supplementary file 1 [file ijms-25-01207-s001.zip › Figure S1.pdf]

A

LMT_vs_HMT

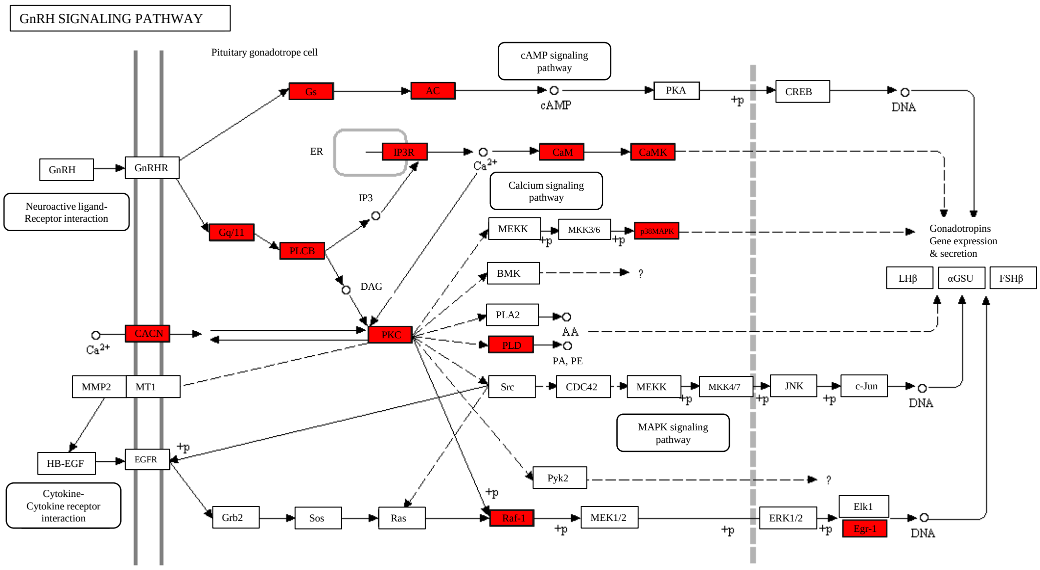

B

LFO_vs_HFO

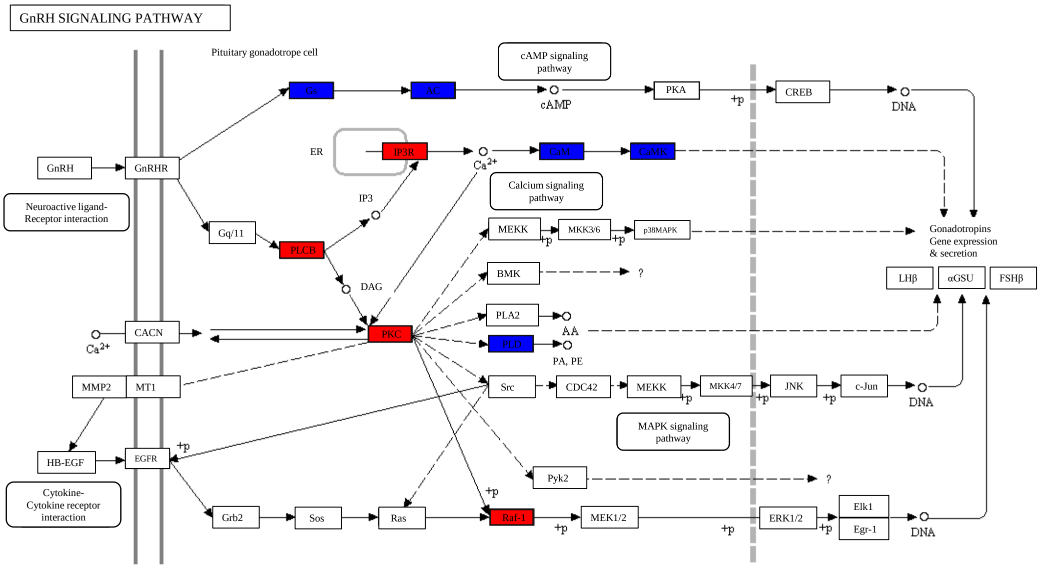

C

LMT_vs_HMT

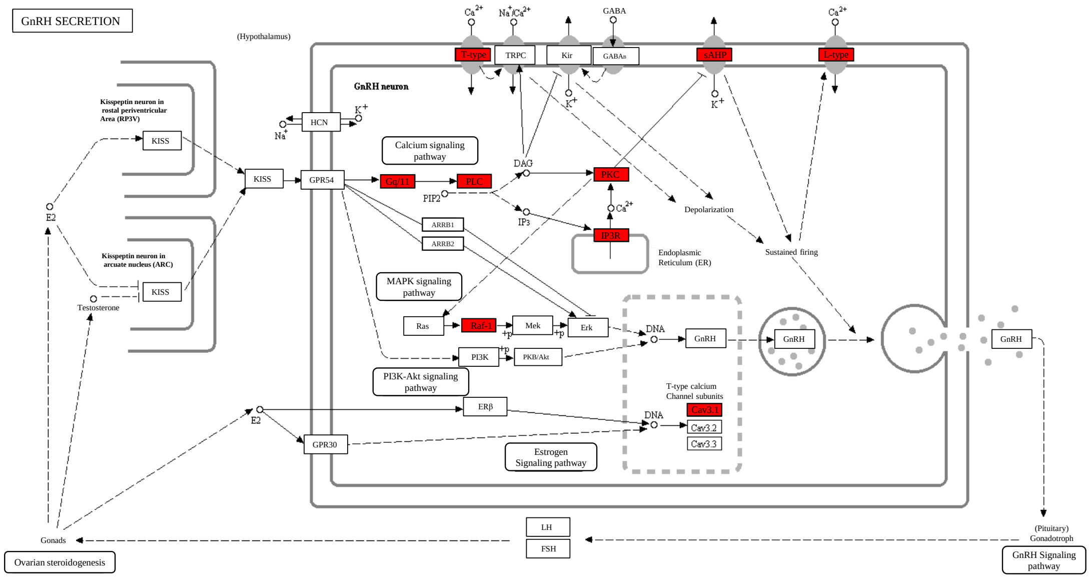

D

LFO_vs_HFO

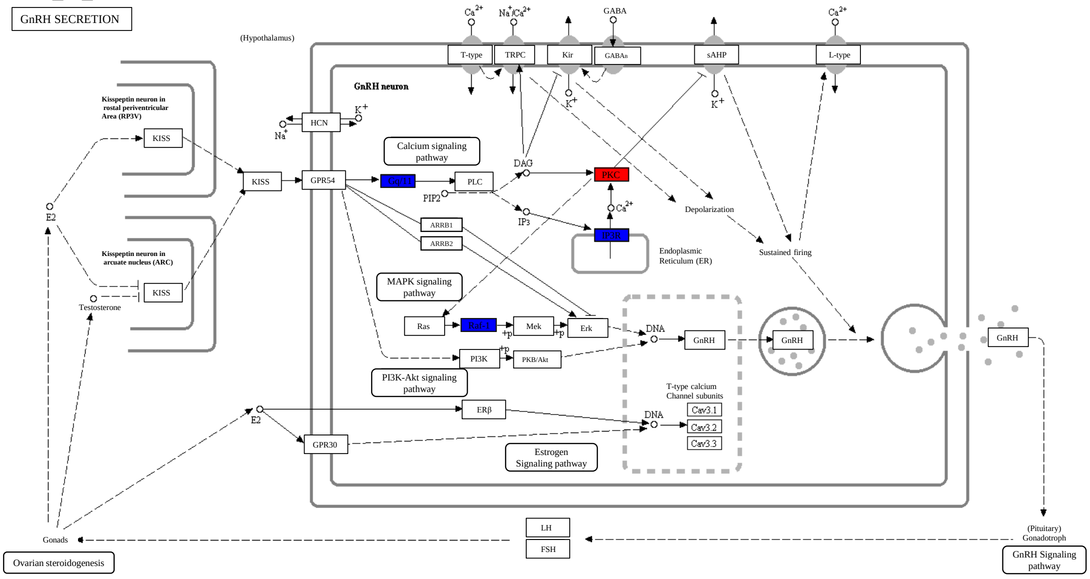

E

LMT_vs_HMT

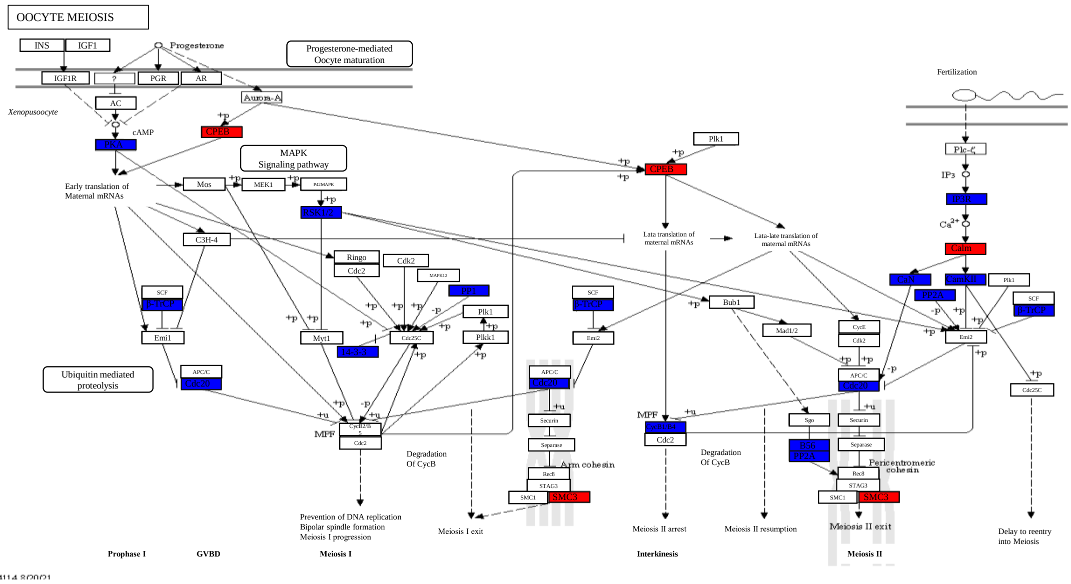

F

LFO_vs_HFO

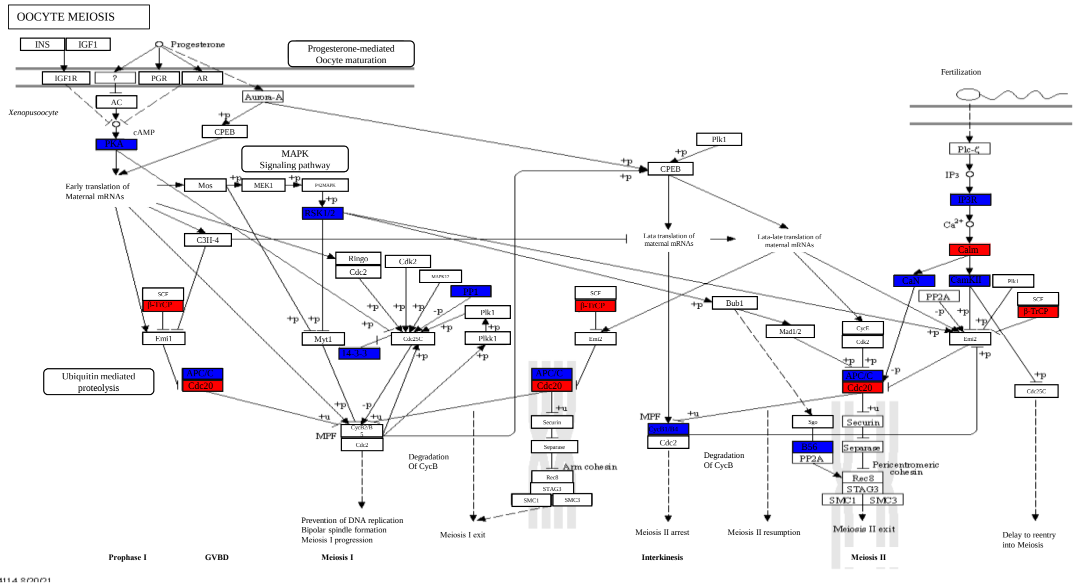

G

LMT_vs_HMT

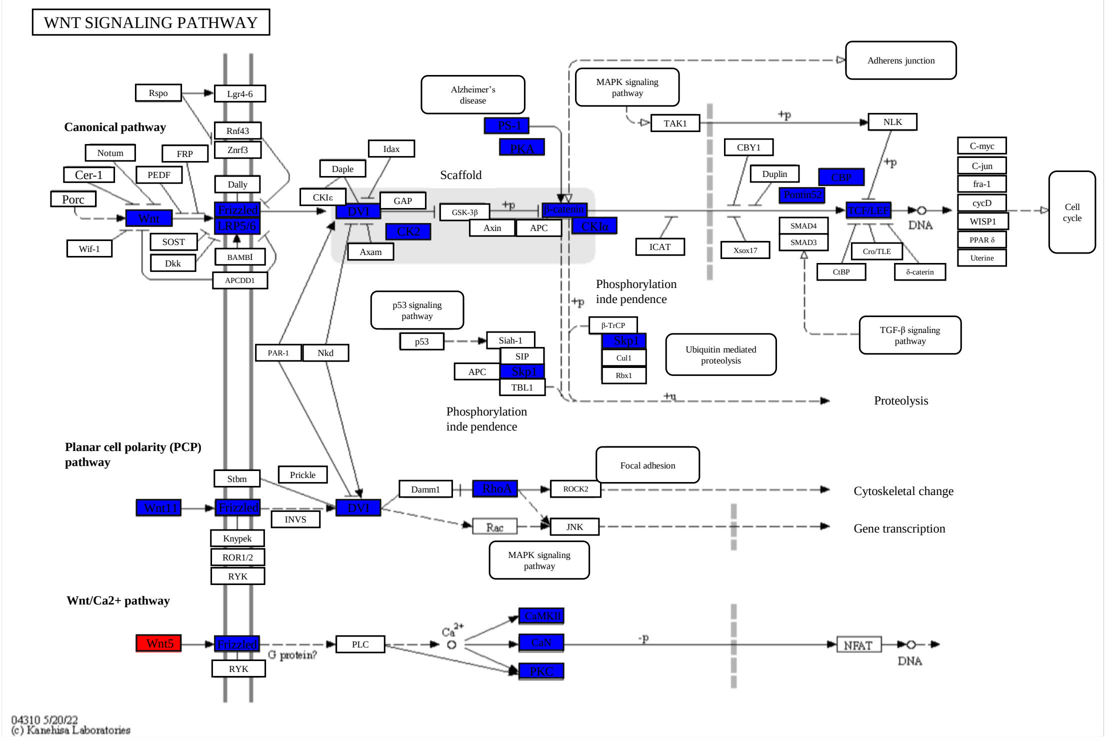

H

LFO_vs_HFO

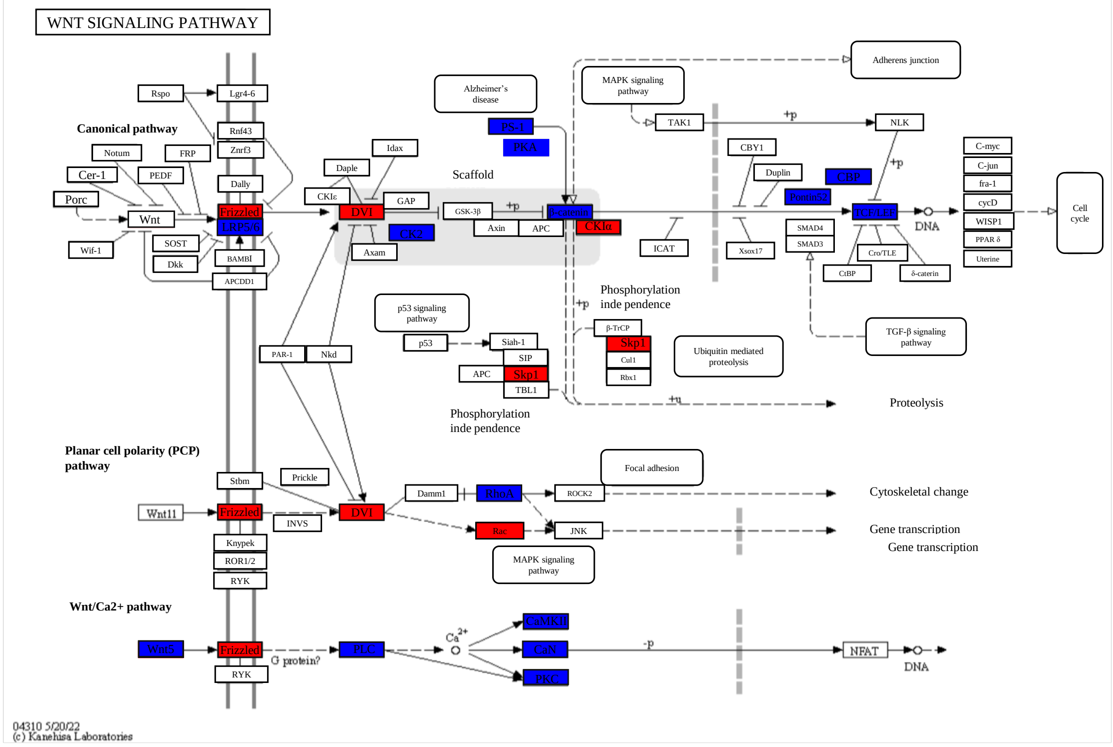

Figure S1. The DEGs involved in signaling pathways related to sex differentiation in *M. nipponense* based on 26 ℃ treatment vs 31 ℃ treatment.
