# Supplementary material for: Temperature-Induced Sex Differentiation in River Prawn (Macrobrachium nipponense): Mechanisms and Effects
Source: Int J Mol Sci. 2024 Jan 19;25(2):1207. doi: 10.3390/ijms25021207 (PMC10816446; doi:10.3390/ijms25021207)
Supplement: Supplementary file 1 [file ijms-25-01207-s001.zip › Figure S2.pdf]

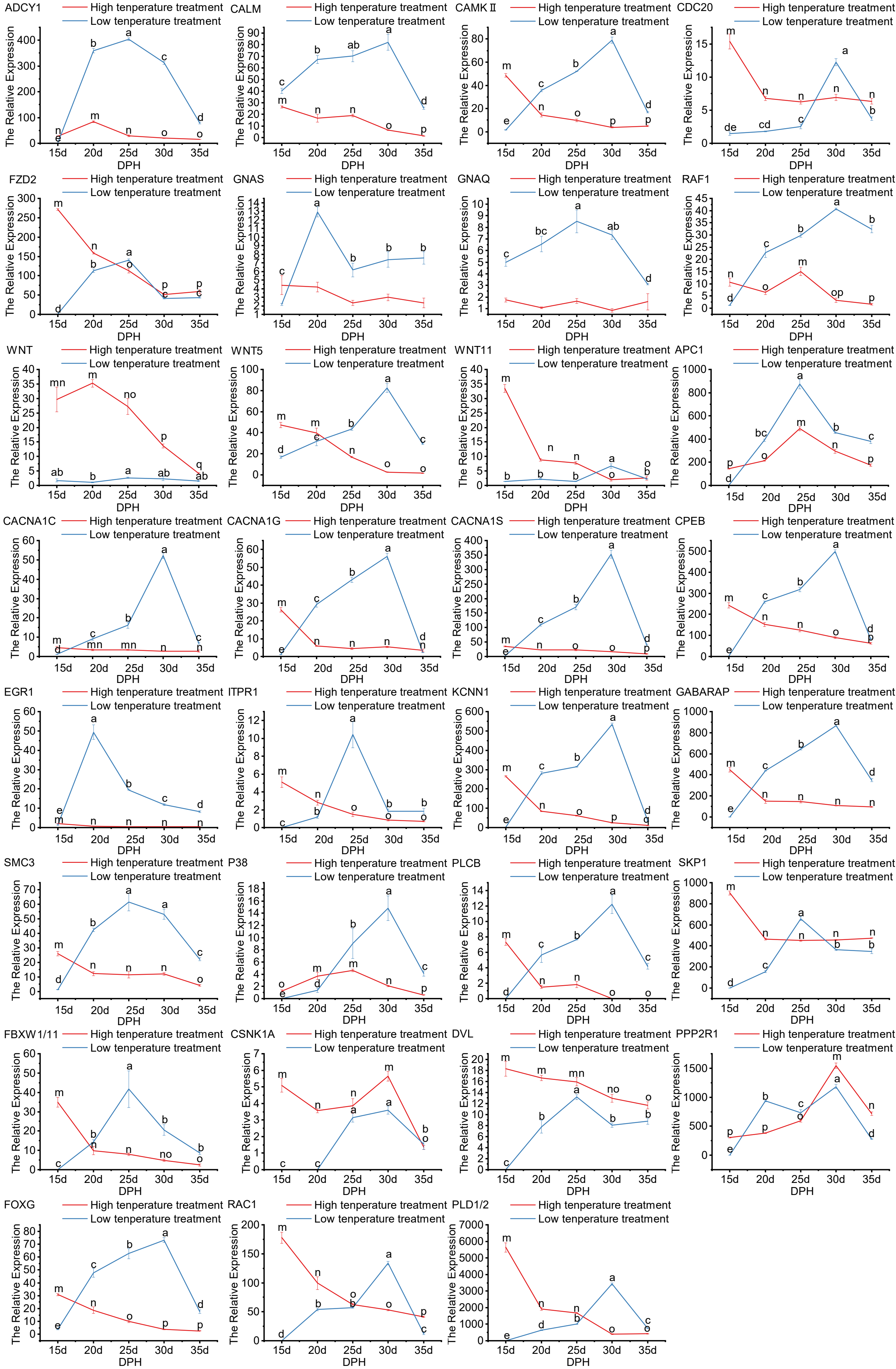
ADCY1
CALM
CAMK Ⅱ
CDC20
FZD2
GNAS
GNAQ
RAF1
WNT
WNT5
WNT11
APC1
CACNA1C
CACNA1G
CACNA1S
CPEB
EGR1
ITPR1
KCNN1
GABARAP
SMC3
P38
PLCB
SKP1
FBXW1/11
CSNK1A
DVL
PPP2R1
FOXG
RAC1
PLD1/2
High tenperature treatment
Low tenperature treatment
The Relative Expression
DPH
15d
20d
25d
30d
35d

Figure S2: The relative expression levels of the temperature-regulated sex-differentiation genes during the sensitive phase of sex differentiation in 26 ℃ treatment and 31 ℃ treatments.
