# Supplementary material for: Temperature-Induced Sex Differentiation in River Prawn (Macrobrachium nipponense): Mechanisms and Effects
Source: Int J Mol Sci. 2024 Jan 19;25(2):1207. doi: 10.3390/ijms25021207 (PMC10816446; doi:10.3390/ijms25021207)
Supplement: Supplementary file 1 [file ijms-25-01207-s001.zip › Table S1.pdf]

Table S1. Primers were used for qPCR verification in this study.

Target gene	Sequence (F:5'-3')	Sequence (R:5'-3')
CACNA1C	GGCGTCTCTGTGCTCCGT	TCCCTCCGCTCCTTCCTT
GNAS	GCGAGTGGTGCTTTAGAG	AGGCAGGGACAGGAGATG
CAMKII	CTCCTTCTTCCAACCTCC	ACTTACCCTGTGCCTCGT
ADCY1	CTTCTTGTTGACTTCGCT	TTTGGTCGTCTCTTCTGT
P38	ATGCCATTTTGCTTGTTG	TTCAGAGGTGATGCGGTT
EGR1	AGTATCCTGCCGCTCGTG	AGGCTGCTTGTTTTCCGA
CALM	GCACAATCACCACCAAAG	AGCCGCAGAGATGAAACC
PLD1_2	ATAATGATGCGGATGAGA	GGTAGAAGGAGGTGAAGG
ITPR1	GGCAATAGACCCACCTTA	GAGCCGTTCCACCACATC
KCNN1	AGTGGGCGTCAGGATGTG	TGTCAGGGTGGAGGGAAT
CACNA1G	ACCTCCAGACATCACCCA	ATTTGACATTAGCCCAGC
PLCB	TCAGGTTGCTGTGTCGGT	CCTTTGAGGGCTTTAGTG
RAF1	CCCCCTACCTTACCTCGT	ACCTCCTGCTGCATACCC
CACNA1S	TCGTCGGTTTCGTTATTG	AGGGAGATGGTGTTGAGC
GNAQ	AGAGTGAGAGCCCCTACA	GCCACCAAGAAAATGATG
FOXG	ACCCCACCCCGCTCCCTT	GGCTGCTGGTTCCGCTCA
GABARAP	AATACAAGGAGGAGCACC	CAGATCACCAATACGAGC
Wnt1	CTGGAAGTGGGGAGGTTG	GCAGAAAAGGAGGGGATG
Wnt5	TGGAGCCCAACAAACAGT	GAGTAAGTCACAACCGTC
Wnt11	GGGAGCGGAGACAGAGAG	TGAAGCCTGAGGCAACAT
DVL	CCAGCCTCAACACACTCA	GGACACCACTCTGCCATT
FZD2	CCGAACTGAGGGAGCACA	CACCCACCAGATCGAGGC
RAC1	TCCTCCTTCAAACACCCA	GGCAACAACCAGTCATCC
SKP1	AATTGATCCTTGCTGCTA	CATTCATTCTCTTTGCGT
CSNK1A	GAAAAAGCACCTCAAATG	AACCGACGACTACAAAAA
FBXW1_11	GAGCATCCTTTATTTGGG	ACAGTCATTGGTGCCGAA
CDC20	TTTTCACCCTCGTGTCGG	GAGTTGCTGCTTTCTGCG
CPEB	TGCGGTAACAACACACTA	CCAGACCATCCACTAAAT

APC1	AATGTTTATTTCTTGCTT	GTGGGTTATTGTTAGTTG
SMC3	TGGAAGAGGATGCTAAAG	GAGAACCCAAATCACAAA
PPP2R1	TACTGCTATCGTTGTGGT	AATAATCTGGAGTTCGGC
β-actin	TATGCACTTCCTCATGCCATC	AGGAGGCGGCAGTGGTCAT
