# Supplementary material for: Temperature-Induced Sex Differentiation in River Prawn (Macrobrachium nipponense): Mechanisms and Effects
Source: Int J Mol Sci. 2024 Jan 19;25(2):1207. doi: 10.3390/ijms25021207 (PMC10816446; doi:10.3390/ijms25021207)
Supplement: Supplementary file 1 [file ijms-25-01207-s001.zip › Table S2.pdf]

Table S2. Summary of the transcriptome sequencing data.

Sample	Total raw reads	Total clean Reads	Q20 (%)	Q30 (%)	GC content (%)
LMT_1	47844874	47743584	98.29	94.54	41.62
LMT_2	46944624	46860098	98.08	94.02	41.43
LMT_3	38008092	37925004	98.14	94.15	41.85
HMT_1	41845280	41781336	97.68	93.11	44.51
HMT_2	46916810	46833500	97.71	93.22	44.97
HMT_3	43408742	43342792	97.72	93.20	44.37
LMM_1	45138996	45026256	98.32	94.87	49.37
LMM_2	44713620	44598182	98.29	94.80	50.22
LMM_3	44483086	44370930	98.31	94.85	50.06
HMM_1	42622042	42559652	97.68	93.48	46.59
HMM_2	43418700	43327050	97.53	93.14	44.59
HMM_3	42641782	42560790	97.51	93.05	45.65
LFO_1	44355232	44257116	98.33	94.61	41.20
LFO_2	47995358	47869586	98.44	94.91	41.22
LFO_3	47656716	47564216	98.29	94.47	41.06
HFO_1	42244110	42189996	97.71	93.13	41.35
HFO_2	42678228	42624286	97.93	93.75	41.34
HFO_3	46627562	46567922	97.88	93.59	41.23
LFM_1	44579566	44476642	98.34	94.95	50.29
LFM_2	41574146	41465346	98.41	95.09	50.02
LFM_3	40727256	40647672	98.35	94.89	50.18
HFM_1	44493944	44381164	97.52	93.10	44.79
HFM_2	40851618	40779098	97.64	93.33	46.06
HFM_3	41843970	41747086	97.48	93.02	45.89
