# Supplementary material for: Temperature-Induced Sex Differentiation in River Prawn (Macrobrachium nipponense): Mechanisms and Effects
Source: Int J Mol Sci. 2024 Jan 19;25(2):1207. doi: 10.3390/ijms25021207 (PMC10816446; doi:10.3390/ijms25021207)
Supplement: Supplementary file 1 [file ijms-25-01207-s001.zip › Table S3.pdf]

Table S3. Summary of the genomic DNA sequencing data.

Sample	Total raw reads	Total clean Reads	Q20 (%)	Q30 (%)	GC content (%)
LFM_1	271467196	266362750	93.47	85.87	26.56
LFO_1	308636428	303633556	95.10	88.60	27.65
LFO_2	269550634	263568422	94.24	87.20	26.94
LFO_3	253443836	245163680	90.48	81.02	28.17
LMM_1	277364906	274455118	94.78	87.46	28.11
LMM_2	259939862	258530778	95.32	88.41	30.84
LMM_3	258146454	256825022	95.36	88.47	30.23
LMT_1	263510448	261847280	96.03	89.72	29.55
LMT_2	282221110	276442194	93.91	86.65	26.75
LMT_3	253759228	247375750	93.18	85.33	25.53
HFM_1	247965518	246685672	95.27	88.32	31.45
HFM_2	266327516	264977678	95.38	88.48	30.91
HFM_3	275583232	273992694	95.32	88.41	30.85
HMM_1	262159602	259130900	94.76	87.57	30.91
HMM_2	284096002	280936004	95.64	89.42	28.64
HMM_3	250628016	249217722	95.02	87.76	30.85
HFO_1	245689636	238718902	92.60	84.54	30.43
HFO_2	257999610	252968560	93.26	85.55	30.48
HFO_3	290664944	286198630	94.16	86.84	33.54
HMT_1	258564004	252491438	92.98	85.19	31.29
HMT_2	257597208	251001922	92.11	83.64	31.46
HMT_3	259057624	249083072	92.07	83.51	31.78
